# The Effect of *Acanthocardia tuberculata* Shell Powder as Filler on the Performance of Self-Compacting Mortar

**DOI:** 10.3390/ma16041734

**Published:** 2023-02-20

**Authors:** Ágata González-Caro, Antonio Manuel Merino-Lechuga, Enrique Fernández-Ledesma, José María Fernández-Rodríguez, José Ramón Jiménez, David Suescum-Morales

**Affiliations:** 1 Área de Química Inorgánica, Escuela Politécnica Superior de Belmez, Universidad de Córdoba, 14240 Córdoba, Spain; 2 Área de Ingeniería de la Construcción, Escuela Politécnica Superior de Belmez, Universidad de Córdoba, 14240 Córdoba, Spain

**Keywords:** self-compacting mortar, seashell powder, waste recycling, sustainable construction, circular economy

## Abstract

In this research, the feasibility of using *Acanthocardia tuberculata* shell waste from the canning industry in the manufacturing of self-compacting mortar (SCM) was tested. The seashells were finely ground to be used as filler instead of the limestone filler normally used in this type of SCM. First, a physicochemical and microstructural characterisation of all raw materials was carried out, including the particle size distribution of both fillers. Subsequently, the self-compactability properties in the fresh state of SCM were evaluated using a total substitution by volume of limestone filler for seashell powder, using different self-compactiblity parameters. The mineralogical phases of all the SCM tested were identified once hardened by means of X-ray diffraction technique, thermogravimetric and differential thermal analysis. In addition, the mechanical properties, water absorption capacity, dry bulk density and accessible porosity of water of hardened mortars at 28 days of curing were analysed. The effect of replacing limestone filler by *Acanthocardia tuberculata* filler resulted in a decrease in compressive strength of 29.43, 16.84 and 2.29%, respectively. The results indicate that it is possible to completely replace natural limestone filler with *Acanthocardia tuberculata* shell filler without significantly affecting the mechanical properties of SCM.

## 1. Introduction

The production of biowaste is a serious problem [1,2]. Seashell waste is a by-product that is considered to be one of the worst environmental problems [3]. Increasing demand and population needs are leading to the growing development of aquaculture production, which in almost 10 years has increased from 58 million tons in 2010 to 85 million tons in 2019 worldwide, according to data from the Food and Agriculture Organisation of the United Nations (FAO). Within the fishery sector, bivalve molluscs (capture plus aquiculture) account for 10% of global production, where China continues to be the main mollusc-producing country. Spain is considered the leading producer of canned fish and shellfish in the European Union, where 86% of production takes place in Galicia [4,5]. The tropical conditions of the Galician estuaries make mussel farming one of the best in the world. Shell waste accounts for 33% of the total weight of mussels, which means that the canning industry generates around 25,000 tonnes of mussel shell waste per year in Galicia [4]. An example of shell waste is the cardid bivalve *Acanthocardia tuberculata* shell (ATS). This type of cardid bivalve is found in southern Spain and on the Atlantic coast of Morocco for the Spanish canning industry [6].

One of the main problems arising in this type of industry, therefore, stems from the management of this by-product, which has little or no industrial value [5]. Some application has been given to increasing the soil pH value in agriculture (as its main component is CaCO_3_), its transformation into calcium supplements for animal feed, handicrafts and jewellery [7]. However, most of it is deposited in landfills, without any treatment. This causes the appearance of gases that are harmful to human health, as a consequence of the decomposition of the organic matter into hydrogen sulphide, ammonia or amines [5]. Therefore, the search for an application for shells would solve a serious problem for the canned fish and seafood industry.

The following concepts (among others) are currently topical research topics: climate neutrality, waste minimisation, moving away from a linear economy towards a circular economy. A very interesting solution for the waste management of shells would be their incorporation as aggregates in mortars or concrete. There have not been many studies that have focused on this task [4,8,9,10,11,12]. This would provide an answer to the topical issues mentioned above.

Concrete is the most widely used building material in the world [13,14]. During its manufacturing process, there are several problems related to the environment, including high CO_2_ emissions and the depletion of natural resources. To manufacture one tonne of ordinary Portland cement, approximately one tonne of CO_2_ is emitted [15,16]. This sector is responsible for the emission of 30% of CO_2_ gases, mainly in the process of transporting and manufacturing materials. Cement production has increased from 2.3 billion tonnes in 2005 to 3.5 billion tonnes in 2020, which also generates a large amount of greenhouse gases [9,11]. As for the use of natural aggregates, the problem is growing, as they are a non-renewable resource usually extracted mainly from quarries or rivers [17]. In several parts of the world, extraction has been restricted due to various floods as a consequence of the change in the course of these rivers, and biodiversity is also affected [18]. Therefore, the use of recycled materials as aggregates (either fine or coarse) or materials that can reduce the use of ordinary Portland cement are essential.

The mortar industry is constantly evolving; one of the greatest advances is the appearance of self-compacting mortar (SCM). The most important characteristic of this is that it flows by the action of its own weight, without the need for vibration energy or any other method of compaction. SCM requires a greater number of fines and the incorporation of superplasticising additives compared to conventional ones, which makes it confer high fluidity properties, giving rise to a finer microstructure, better durability and mechanical properties [19,20]. Because the demand in the use of building materials is increasingly high due to urban growth and the industrial sector, it is necessary to look for sustainable alternatives, both to avoid problems related to the consumption of natural resources and environmental pollution. There have been many research studies on the multitude of waste used in mortar. The recovery of this waste is one of the most important issues to achieve an adequate circular economy [21,22].

The use of seashells from canning industries as a substitute for conventional aggregates, cement or filler in concrete could be a good alternative, both to reduce the consumption of natural aggregates and thus to avoid the depletion of these resources and CO_2_ emissions, as well as to reduce their accumulation in landfills. There have been some studies that have used shells from different species of molluscs. The results obtained in each of the studies have depended on the percentage of substitution used and the type of shell (mussel, oyster, cockle, scallop, etc.) and the additions and additives used. All of the studies concurred that the shells are composed of 93% calcium carbonate or higher, which is very positive when substituting them for conventional materials [5,7,23]. 

Martinez et al. [4] studied the behaviour of mussel shells, substituting them for coarse and fine aggregates in plain concrete. The mechanical properties of the mortars worsened with the percentage of substitution. This fact was due to the higher water absorption of the shells, their shape and the presence of organic matter, which was removed with heat treatment by several authors [9,10,24]. The most important problem when using shells derives from both the shape and the porosity of the particles, which leads to higher water absorption. Several authors [5,23] have studied the water requirements of different types of shells, mainly focusing on both the shape and the porosity of the particles, which is also the main consequence of the worsening of mechanical and workability properties [7].

The aim of this work is to study the effect of replacing limestone filler (LF) with filler from ATS to test the viability of this type of waste in SCM production to achieve a double objective: reduce the accumulation of this waste in landfills, giving them a second useful life and avoiding the consumption of non-renewable natural resources, thus promoting the new paradigm of the circular economy in the construction sector. No studies have been found using ATS powder as a filler replacement for SCM.

Firstly, a complete physicochemical and microstructural characterisation of all raw materials was carried out. In order to check the effectiveness of the heat treatment performed on the shells, a microstructural analysis by SEM was carried out on the outer part of a shell. The effect on the fresh and hardened properties of the SCM tested was studied, as well as the X-ray diffraction technique, thermogravimetric and differential thermal analysis of all hardened mortars, in addition to analysing the mechanical properties, water absorption capacity, dry bulk density and accessible porosity of water of hardened mortars at 28 days of curing. 

## 2. Materials and Methods

### 2.1. Materials

In this research the following materials were used: two types of natural sand (fine sand 0/3, FS, and coarse sand 0/6, CS) and a commercial limestone filler (LF) from Taljedi, S.L. The cement was CEM II A/L 42.5 R [25]. In order to maintain consistency, a superplasticiser additive (Sp), MasterEase 5025 from the Master Builders Solutions S.L.U. company, was used, composed of Poly-Aryl-Ether polymers that are especially indicated for highly fluid, self-compacting and high-strength concrete.

ATS powder was used as seashell filler (SF) in order to find out the effect in the properties of SCM, obtained from the Grupo Ubago company in Málaga, Spain. To obtain SF from ATS waste, a previous milling/mechanical treatment was necessary to significantly reduce the particle size.

In this research, CEM, LF and SF were considered powder materials (Vp) and FS and CS were considered fine aggregates (Vs).

### 2.2. Pre-Treatment of Acanthocardia tuberculata

A heat treatment before grinding the ATS waste was necessary to remove organic material and bacteria. The heat treatment was carried out according to European Standard 1069/2009 [26] using a laboratory oven at 135 °C for 32 min to ensure that the use of such shells was not harmful to health [4]. The sulphate concentrations for ATS were 0.05 and 0.01% in mass (UNE-EN 1744-1) before and after of washing. These values were lower than the limit specified by Structural Code [27]. Regardless of this, the shells were washed to remove dirt from them.

**Figure 1:**
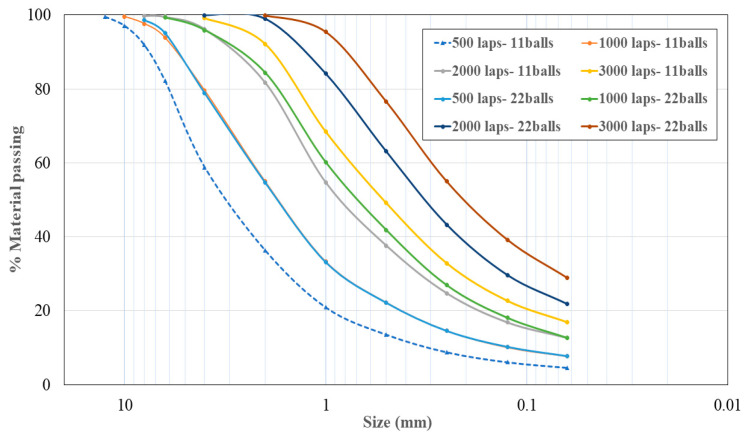
Particle size distribution of *Acanthocardia tuberculata* shells (ATS) with different grinding processes.

To grind the ATS once treated, a Los Angeles machine was used as a mill. Eight types of milling were performed, with 11 and 22 standardised steel balls, modifying the number of laps. The particle size distribution curves of the different grindings were obtained according to UNE EN 933-1: 2012 [28]. Figure 1 shows the particle size distribution of the ATS powder shells. The number of laps studied was from 500 to 3000, combined with 11 and 22 balls. 

According to the Spanish structural code [27], the aggregates that pass through a 0.125 mm sieve are considered as filler. The chosen grinding procedure was 3000 laps combined with 22 balls, because the amount of material with a size less than 0.125 mm was greater. In order for both fillers (LF and SF) to have a similar particle size, the grinding material was passed through a 0.125 mm sieve before being used in the SCM mixtures. Using this procedure, only 40% of SF can be obtained. In further research, the remaining 60% of SF will be used as fine aggregates. In this study, only this fraction was studied (as filler).

### 2.3. Self-Compacting Mortar Design

To obtain adequate self-compacting properties, an experimental and iterative process was carried out, based on both the Nepomuceno method [28] and the European Federation of National Associations Representing producers and applicators of specialist building products for Concrete (EFNARC) [29], on six types of SCM with the following ratios: absolute volume of powder materials and fine aggregates (Vp/Vs); absolute volume of water and powder materials (Vw/Vp); percentage between the amount in mass of superplasticiser and powder materials (Sp/p %).

Three types of SCM (Ref-1, Ref-2 and Ref-3) were elaborated varying the ratio (Vp/Vs) from 0.6 to 0.8 at 0.1 intervals, which were used as reference. The w/c ratio was fixed for all mixtures at 0.49. The (Vw/Vp) ratio remained constant with a value of 0.85 and the percentage between the amount in mass of superplasticiser and powder materials (Sp/p %) ranged from 0.5 to 0.7 at 0.1 intervals. A replacement volume of 100% SF was tested for Ref-1, Ref-2 and Ref-3. The density of LF and SF was measured using a helium pycnometer. Very similar densities were obtained, 2654 and 2613 kg/m^3^ for LF and SF, respectively. Table 1 shows nomenclature, proportions used for each mixture (kg/m^3^) and parameters of the SCM.

The relative spread area (Gm) and the relative flow velocity (Rm) parameters were measured through slump-flow and v-funnel tests, respectively, to check if the mortar mixtures (Table 1) met the self-compacting conditions. The adequate values were those that fulfilled the flow requirements experimentally measured with the above two tests; hence, the (Sp/p %) ratio was modified in each mortar mixture (Table 1). Values of Rm and Gm were calculated according to Equations (1) and (2), respectively, where Dm stands for the average spread diameter, in mm; D_0_ stands for the initial diameter at the base of the cone, 100 mm; and t stands for the time of flow, in seconds.

(1) $$\mathrm{Gm}={\left(\;\frac{\mathrm{Dm}}{D_{0}}\right)}^{2}-1$$

(2) $$\mathrm{Rm}=\frac{10}{t}$$

All mixtures to achieve SCM were developed with the same kneading procedure. First, the dry materials (maintained at 105 °C degrees for 24 h) were homogenised without water at low speed for 1 min. The water was added to the superplasticiser and mixed. After that, homogenised dry materials were added to the water and superplasticiser and mixed for 6 min at low speed. The mixture was kept at rest for 2 min and finally mixed again for 2 min at low speed. 

For each type of mortar, prismatic moulds (40 mm × 40 mm × 160 mm) were made [30]. After 24 h, each of them was unmoulded and cured at 95 ± 5% relative humidity and 20 ± 2 °C temperature until 28 days. 

### 2.4. Tests Methods

#### 2.4.1. Characterisation of Raw Materials and Hardened Mortars

- X-ray fluorescence spectrometry analysis (XRF)

To characterise the chemical composition of raw materials (CEM, LF, SF, FS and CS), XRF was performed with a power of 4 kW and ZSX Primus IV (Rigaku) equipment.

- X-ray diffraction pattern (XRD)

The raw materials and hardened mortar mineral phases were identified using XRD patterns. A Bruker D8 Discover A25 instrument with CuKα (λ = 1.54050 Å; 40 kV; 30 mA) was used; the diffraction pattern was obtained by scanning the goniometer from 10° to 70° (2θ) at a rate of 0.006 θ min^−1^. Hardened mortar samples were prepared by grinding them to a powder, after being immersed in ethanol for 48 h, to obtain a representative sample of each material.

- Determination of the particle density of filler

The density of LF and SF at atmospheric pressure was measured with a gas pycnometer (MICROTRAC BELPycno Ver 1.14 L) using helium gas. Three samples of each type of filler were tested.

- Thermogravimetric analysis and differential thermal analysis (TGA-DTA)

Thermogravimetric analysis (TGA) and differential thermal analysis (DTA) were performed for the raw materials and hardened mortar samples. TGA was performed in a Setaram Setsys Evolution 16/18 apparatus at a heating rate of 5 °C·min^−1^, the testing temperature ranged from room temperature to approximately 1000 °C.

- Scanning electron microscopy (SEM)

According to the European Standard 1069/2009 [26], seashells must be heated at 135 °C in order to eliminate the bacteria. An analysis using SEM was carried out on an ATS, heated and unheated, to check this behaviour. To obtain the maximum quality in the images, the samples were sputtered with gold. Furthermore, the microstructure of the fillers obtained was also studied.

#### 2.4.2. Fresh Properties of the Mixtures

The slump flow and v-funnel test were used to measure the self-compactability properties. According to the European Federation of National Associations Representing producers and applicators of specialist building products for Concrete (EFNARC) [29], the cone of the slump flow test must have a diameter of 100 mm, and the v-funnel test was used to determine the flow time of the SCM with a height of 300 mm.

#### 2.4.3. Hardened Properties of the Mixtures

- Flexural and Compressive Strength

Mechanical properties of the mixtures were measured using flexural and compressive strength after 28 days of curing at 95 ± 5% of relative humidity and 20 ± 2 °C temperature, according to the European Standard EN 1015-11 [30]. Three samples of each type of mixture were tested for flexural strength (prismatic 40 mm × 40 mm × 160 mm) and six samples for compressive strength (semi-prismatic 40 mm × 40 mm × ≈ 80 mm).

- Dry bulk density, water absorption capacity and accessible porosity of water

The dry bulk density, water absorption capacity and accessible porosity of water of hardened mortars were determined according to the European Standard UNE 83980 [31]. Two samples of each type of mortar were tested at the age of 28 days.

## 3. Results and Discussion

### 3.1. Effect of Thermal Treatment in the Acantocardia tuberculata

The microstructure of ATS, before and after heat treatment, was studied by SEM. Several previous studies have focused on studying the microstructure of these seashells [32,33,34,35]. However, there is no research on the effect of heating on the microstructure of the seashells. This section attempts to present the most notable changes. 

**Figure 2:**
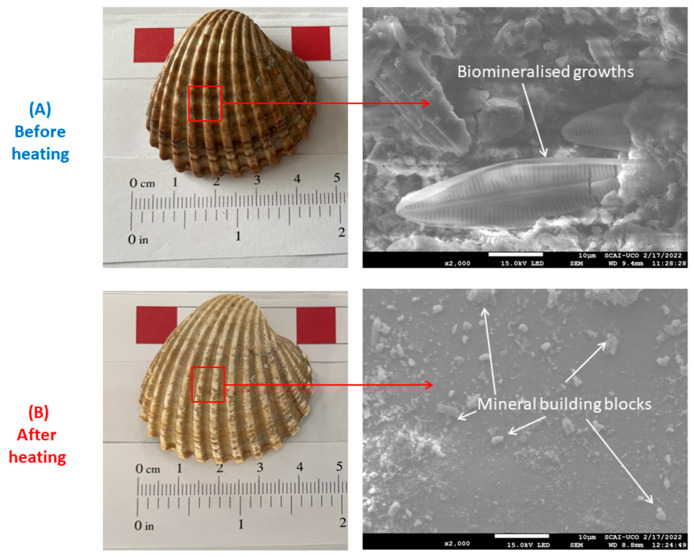
Image before (**A**) and after (**B**) heat treatment of *Acanthocardia tuberculata* shell and SEM observations. Biomineralised growths.

**Figure 3:**
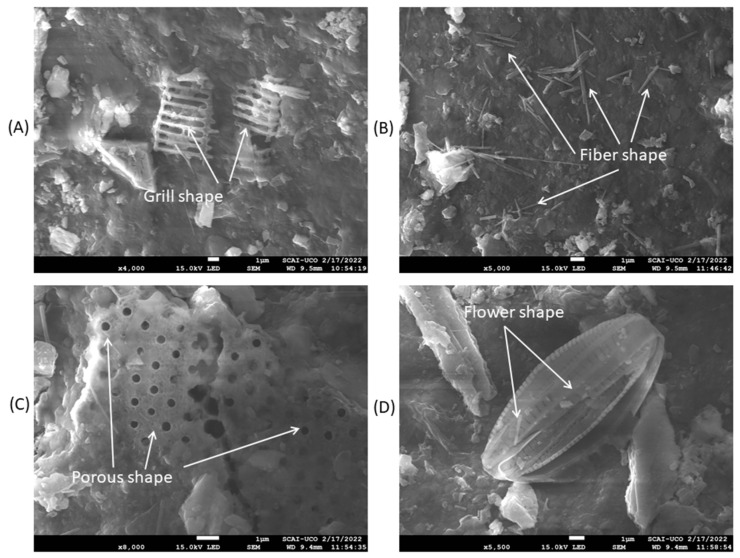
Different “sophisticated shapes” resulting from the biomineralisation of the shells on the outside before heating: (**A**–**D**).

Figure 2 shows images of the appearance of the ATS shell itself before and after heating (left side) and SEM images at high magnification (right side). It should be noted that no differences were found at low magnifications. However, significant differences were found at high magnification. On the outside of the unheated shell, a complex, repeatedly “sophisticated shaped” form was observed. In addition, other types of complex structures were also found (Figure 3). This was the result of the biomineralisation of seashells (with an incredible diversity of macro-and micro-architectures) [35,36]. These biomineralised materials are usually minerals (e.g., CaCO_3_) and organic macromolecules (e.g., protein and polymer) [37]. Other shaped tips were also found, as presented in Figure 3 (before heating). On the heated shell, a smooth surface was observed, and on this surface mineral, building blocks were observed; very usual in these microstructures [35].

Heating the shells before use leads to the removal of the biomineralisation structures, as proven by SEM. This can lead to a worsening of the mechanical properties of ATS. In the structure of seashells, only a few percent of organic macromolecules are present, yet impressively, these materials exhibit toughness at orders of magnitude higher than their mineral phases (e.g., CaCO_3_ or their polymorphs) [33,38,39]. For future research, it may be beneficial to avoid heating ATS. This should be checked with a leaching analysis, which shows that this residue is not hazardous to the environment, as was determined in previous research by the authors [40].

### 3.2. Characterisation of Raw Materials

Table 2 summarises the chemical composition of raw materials obtained from XRF analysis, expressed in percentage of oxides. The largest chemical oxide composition for all raw materials (CEM, FS, CS, LF and SF) was CaO. The higher CaO content in FS than in CS may indicate a higher “purity” of FS. The LF and SF reflected a very similar composition of CaO. These indicate the feasibility of replacing LF by SF, regarding their chemical composition. The CaO content obtained in SF was higher than that found by other authors; the percentage of CaO depends on the type of shells and thermal treatment; when shells are subjected to heat, partial decomposition of calcite occurs, which forms CaO and carbon dioxide [7]. In oyster shells, the CaO content found by other authors was: Yang et al. 51.06% [41]; Lertwattanaruk et al. 53.59% [23]; Yoon et al. 59.94% [42], which were heated to around 105 °C. Li et al. [43] and Djobo et al. [44] calcined oyster shells to temperatures of 850–950 °C and 500 °C, respectively, and the CaO content obtained was 86.80% and 74.73%, respectively. The CaO content found in mussel shell was: Lertwattanaruk et al. 53.38% [23]; Yao et al. 53.70% [45] and Felipe-Sese et al. 87.21% [46]. The latter calcined the shells at 1100 °C. In cockle shell, CaO content was: Lertwattanaruk et al. 54.24% [23]; Olivia et al. 51.56% [47]; Olivia et al. 51.9% [12].

**Figure 4:**
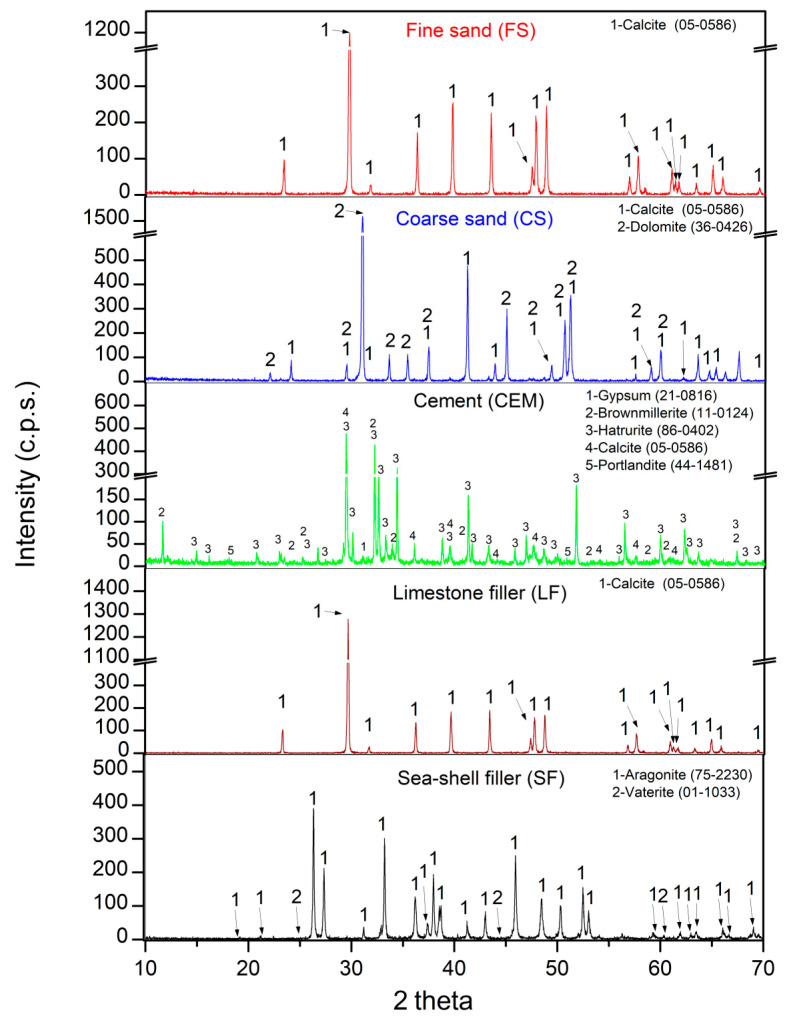
XRD diffraction of Fine sand (FS), Coarse sand (CS), Cement (CEM), Limestone filler (LF) and Seashell filler (SF).

Figure 4 shows the XRD patterns of all raw materials used. For FS, the main phase found was calcite (05-0586) [48] and the same phase was found in LF. The phases found in CS were calcite (05-0586) [48] and dolomite (36-0426) [48]. The “purity” of FS versus CS was already indicated in the XRF results, where CaO content was higher for FS than for CS (which is why the dolomite phase appears in CS, in accordance with the MgO content found in Table 2). The phases found in SF were aragonite (75-2230) [48] and vaterite (01-1033) [48]. Both phases are polymorphs of CaCO_3_, and have featured often in different research papers [36,39]; the formation of these polymorphs is linked to the biogenic origin of the shell [8,33]. The tipycal phases of cement [49,50] were also identified. 

**Figure 5:**
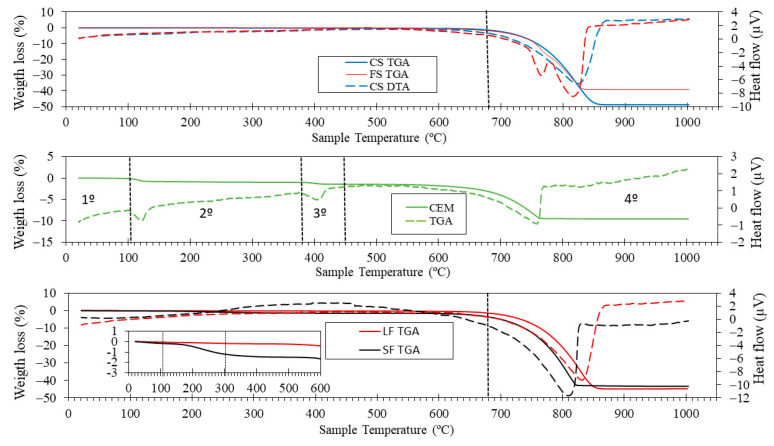
Thermogravimetric analysis (solid lines) and differential thermal analysis (dotted lines) for all the raw materials.

Figure 5 shows the thermogravimetric analysis (TGA) and differential thermal analysis (DTA) for all the raw materials. For FS and CS, practically no weight loss was observed until about 680 °C. This is in accordance with the fundamental phase found in XRD for both types of sands (calcite), which starts to decompose from this temperature onwards. Similar results have been found by Suescum-Morales et al. [51,52]. For cement, four stages were observed: the first is attributed to moisture loss; the second to aluminates and silicates decomposition [20]; the third to portlandite [17]; and the fourth to calcium carbonate decomposition [13,53].

For LF, no weight losses were found up to 680 °C. This is in accordance with the calcite phase found in XRD. However, for SF, the behaviour was somewhat different, as can be observed in the inset of Figure 5 SF showed a characteristic TGA profile observed in other kinds of shells [4,8,54]. Several stages were observed: (i) from room temperature, up to 105 °C humidity was lost; (ii) from 105 to 300 °C, the organic matrix was lost (cellular structure of the mollusc) [55]; (iii) from 300 to 600 °C, the weight loss was practically non-existent, although in DTA a peak appeared at approximately 500 °C. This would correspond to specific organic compounds with significant presence, whose sublimation temperatures were reached [4]. (iv) From 600 to 1000 °C, calcium carbonate decomposition occurred [1,56]. In addition, a delay in the endothermic peak was observed for SF (it occurred at approximately 810 °C), while, for LF, it occurred at 830 °C. Similar behaviour was observed by J.Wang et al. [57].

**Figure 6:**
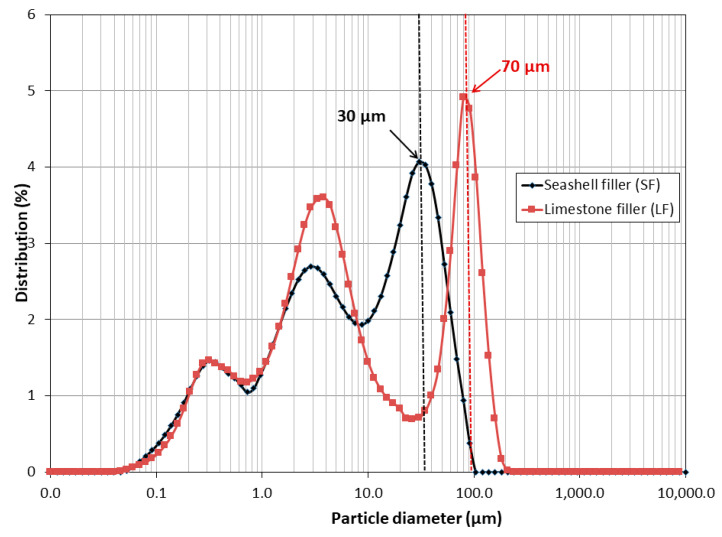
Particle size distribution of Seashell filler (SF) and Limestone filler (LF).

Particle size distribution for LF and SF is shown in Figure 6. The particle size of LF ranged between 0.06 µm and 200 µm, and between 0.06 µm and 100 µm for SF. Both fillers had a particular trimodal size distribution. The highest percentage of particles was 70 and 30 µm for LF and SF, respectively.

**Figure 7:**
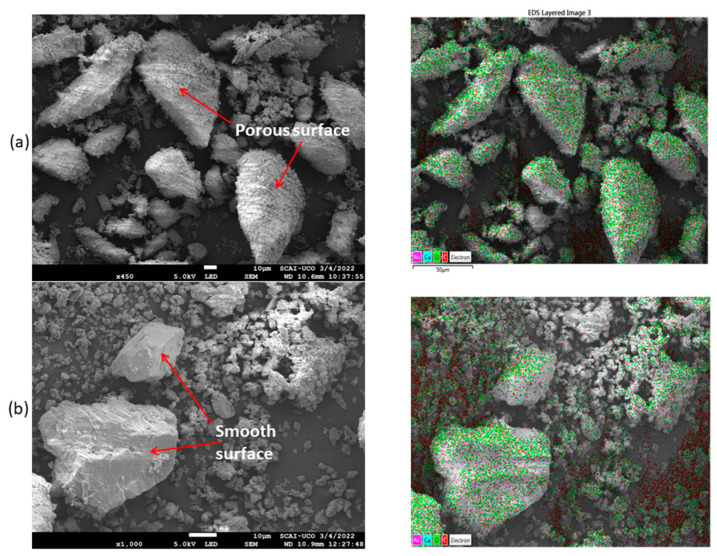
SEM images of (**a**) Seashell filler (SF) and (**b**) Limestone filler (LF).

Figure 7 shows the SEM micrographs of SF (a) and LF (b). For both samples, different particles sizes were found. This confirms the results found regarding the particle size distribution (Figure 6). SF particles appear less smooth and more porous than LF particles. A very similar result was obtained by Rojo-López et al. [58] for a similar LF. No SEM images of ATS particles have been found with a procedure similar to the one used in this study. The mapping result shows that the main elements were Ca and O for both elements, which is in accordance with the XRF results obtained (Table 2). Au and C were the result of the coating and carbon tape, respectively.

### 3.3. Fresh Properties of the Mixtures

Table 3 shows Gm and Rm parameters obtained for each of the SCM studied. The admissible interval was between 4.29 and 7.41 for Gm, and 0.83 and 2.50 s^−1^ for Rm. An experimental and iterative procedure was carried out, modifying the Sp/p% ratio, until SCM that met the Gm and Rm parameters recommend by Nepomuceno [28] and the specifications of EFNARC [29] were obtained. The values obtained were admissible due to none of the mixtures showing segregation or bleeding. Therefore, three types of mixtures with 100% SF from ATS that meet the parameters of self-compactability as SCM are presented.

The need to add Sp (Table 1) was higher when SF was incorporated into the mixtures, since the fluidity (Rm) and consistency (Gm) of SF mixtures decreased with seashell powder incorporation. This behaviour may be related to particle size distribution (Figure 6), as LF particles are larger than SF particles. Furthermore, SF particles were more porous and had a less smooth surface of SF than LF (Figure 7). This may explain why the mortar with SF absorbs more water than mortar with LF. If the water content is changed, properties such as fluidity, cohesion or passing ability can be modified in SCM. For this reason, the Vw/Vp ratio remained constant in all batches (Table 1), and the Sp was increased. This behaviour is in accordance with the previous experiments of other authors, who agreed that it is related to the shape and surface texture of the particle. Lozano-Lunar et al. studied self-compacting properties in SCM, replacing siliceous filler with two types of wastes: granite sludge and electric arc furnace dust as filler, and concluded that when there was an increase in the substitution percentage of both wastes, Rm and Gm values decreased. This behaviour was also attributed to the surface texture and particle size distribution, and the Sp/p ratio had to be increased [19,59]. Benabed et al. [60] and Bosiljko et al. [61] studied self-compactability properties when substituting limestone filler with sand and Portland cement, respectively. Both agreed when the percentage of limestone filler increases, the slump flow decreases and v-funnel flow time increases, due to a larger specific surface and the fineness of limestone filler, which requires more water. Safi et al. [62] studied the ability of self-compactability properties to use seashells as fine aggregate (with sand substitution) in SCM. The same results as [60,62] were obtained for slump flow and v-funnel flow; as the percentage of substitution increases, the workability becomes worse, and the effect of shells on the workability of SCM is attributed to the irregular shape of the particles. This causes greater friction between particles and an increase in the surface area, leading to greater water absorption, since more internal voids are formed.

### 3.4. Hardened Properties

#### 3.4.1. Characterisation of Hardened Mortar

**Figure 8:**
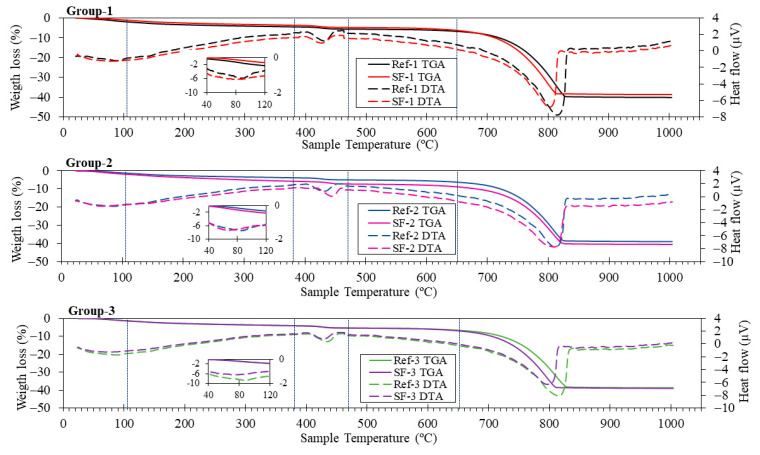
Thermogravimetric analysis (solid lines) and differential thermal analysis (dotted lines) for all groups of mixtures.

Figure 8 shows the TGA/DTA results for different groups of mortar mixtures. There are five main temperature ranges in TGA/DTA curves, namely room temperature—105 °C; 105–380 °C; 380–470 °C; 470–650 °C and 650–1000 °C. 

In the first stage, moisture (physically absorbed), ettringite and C-S-H were lost (which was related to the slight endothermic peak observed in the inset for all samples) [57]. The losses occurring in the second stage (105 to 380 °C) were attributed to the decomposition of hydrated calcium silicates and aluminates [20]. Between 380 and 470 °C, a peak appeared due to the decomposition of portlandite (Ca(OH)_2_) [13,63]. Note that the peak was centred at different temperatures, depending on whether the mixture was Ref (1, 2 or 3) or SF (1, 2 or 3). This may indicate that the portlandite comes from different sources: either from the hydration of the cement, or from SF (already observed with TGA/DTA). After this, the Ca(OH)_2_ content was calculated, using the weight loss due to the dehydroxilation of portlandite. The weight percentage was 1.10, 1.14, 1.17, 1.59, 1.29 and 1.31% for Ref-1, SF-1, Ref-2, SF-2, Ref-3 and SF-3, respectively. Therefore, it can be stated that the amount of Ca(OH)_2_ increases with the use of SF from ATS. A similar result was obtained by J. Wang et al. [57]. These authors indicated that the increased portlandite formation was due to the physical function of the seashell dust. However, it was not the same type of shell, although the microstructure and composition were very similar. Further research will be needed to definitively establish this claim.

From 470 to 650 °C, the initial carbonates formed in the hardening process of the cement were lost [64]. From 650 to 1000, the decomposition of the calcium carbonate took place [51,52]. It should be noted that for the mixtures containing SF there was a delay in the endothermic peak, which was already observed in the characterisation of the raw materials (Figure 5).

**Figure 9:**
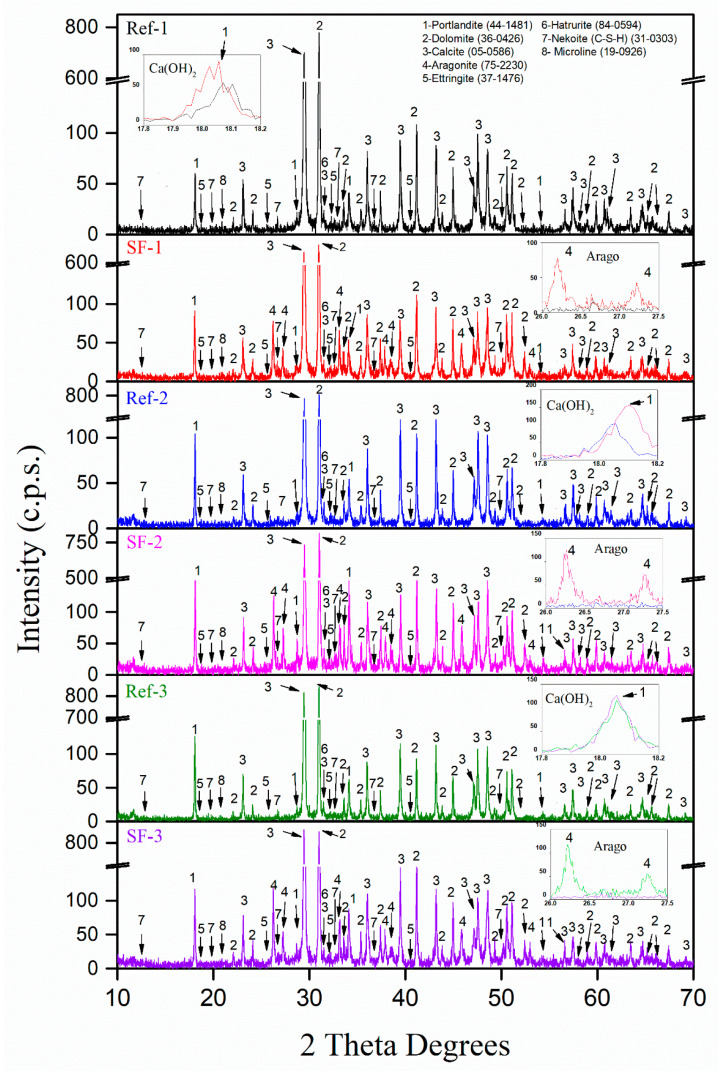
XRD diffraction of all reference and seashell filler (SF) mixtures.

Figure 9 shows the XRD patterns of all Ref (1, 2 and 3) and SF (1, 2 and 3) mortar mixtures obtained after curing at 28 days. The main phases identified for Ref-1 were: Calcite (CaCO_3_) (05-0586) [48] and Dolomite (CaMg(CO_3_)_2_) (36-0426) [48]. These phases come from FS, CS and LF used for this mixture (see Figure 4). Clearly, the Portlandite phase [Ca(OH)_2_] (44-1481) [8] was also found. This was attributed to the presence of this phase in the cement itself (see Figure 4) and to the hydration reactions of hatrurite (also named alite) in the cement itself (3) [65,66]:

(3) $$2\left(3\mathrm{CaO}\cdot {\mathrm{SiO}}_{2}\right)+6H_{2}O\to 3\mathrm{CaO}\cdot 2{\mathrm{SiO}}_{2}\cdot 3H_{2}O+3\;\mathrm{Ca}{\left(\mathrm{OH}\right)}_{2}$$

The Ettringite phase (Ca_6_Al_2_(SO_4_)_3_(OH)_12_·26H_2_O) (37–1476) [48] was also found. This phase appears when the hatrurite is mixed with the gypsum together with water, as described in (4) [65,66]:

(4) $$3\mathrm{CaO}\cdot {\mathrm{Al}}_{2}O_{3}+3{\mathrm{CaSO}}_{4}+32H_{2}O\to 3\mathrm{CaO}\cdot {\mathrm{Al}}_{2}O_{3}\cdot 3{\mathrm{CaSO}}_{4}\cdot 32H_{2}$$

The presence of a calcium silicate hydrate called Nekoite (Ca_3_Si_6_O_12_(OH)_6_·5H_2_O) (31-0303) [48] was also confirmed. This is the widely known result of the hydration of the hatrurite phase. However, it seems that the Hatrurite phase 3(CaO·SiO_2_) (84-0594) [48] was not completely hydrated, as very small peaks were found in the diffractogram. This was normal, as the samples were only obtained after 28 days of curing. Microline (KAl_3_SiO_8_) (19-0926) [48] was also found. This was result of chemical reactions in the cement used [19]. All of these results are in accordance with the phases found in TGA/DTA for Ref-1.

In each XRD pattern, the intensity of the characteristic peak for anhydrous or hydrated paste approximately indicate the “amount” of that phase [67]. The same phases found for Ref-1 were found for SF-1. This was indicative that the presented SF has no negative aspects on cement setting, which would occur with significant changes in XRD. However, the Aragonite phase (CaCO_3_) (75-2203) [48] was found, which is in accordance with the main phase of the SF used. This can be seen in the inset labelled “Arago”, zooming in between 26 and 27.5 2 theta (line red and black). In addition, an increase in the relative intensity of Ca(OH)_2_ was observed between the Ref-1 and SF-1 mixtures, shown in the inset labelled “Ca(OH)_2_” (line red and black). This could be due to the high CaO content found in XRF of SF, as described by G.O. Bamigboye et al. [2]. A.Hasnaoui et al. [54] confirmed, in a preliminary study with alkaline activated materials, that the use of seashells increased the portlandite content. A.Naqi et al. [68] also confirmed the increased portlandite content with calcined oyster shell, but in this case using the TGA/DTA technique. This observation of the increase in portlandite with the use of SF has already been indicated (and calculated) with TGA/DTA.

The same phases were found for Ref-2 as for Ref-1, which indicated that the increase in the Vp/Vs ratio from 0.6 to 0.7 does not affect the mineralogical composition of SCM. Again, comparing the use of LF with SF (i.e., Ref-2 vs. SF-2 mixtures) a higher relative intensity of the portlandite phase was found (see inset “Ca(OH)_2_” comparing the blue and pink lines). Furthermore, the use of SF made it present the Aragonite phase in SF-2. The same behaviour as for Ref-2 was obtained for Ref-3. For the SF-3 mixture, a higher intensity of portlandite was obtained (a little lower than in the previous cases). Note that the difference between the two was 1.29 and 1.31% (calculated with TGA/DTA), which may explain why the difference is so slight.

Again, the Aragonite phase was found (see the green and purple lines inset). No studies were found that performed the substitution of LF for SF (from ATS) for SCM, or that were also analysed by XRD.

#### 3.4.2. Flexural and Compressive Strength 

**Figure 10:**
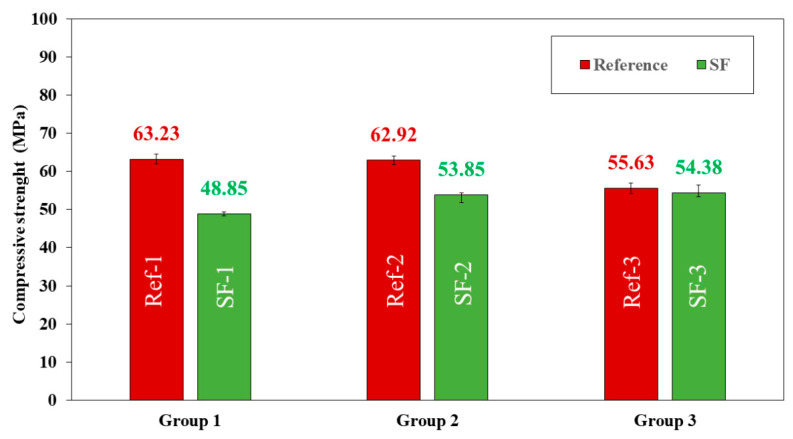
Compressive strength of all reference (Ref) and seashell filler (SF) mixtures.

**Figure 11:**
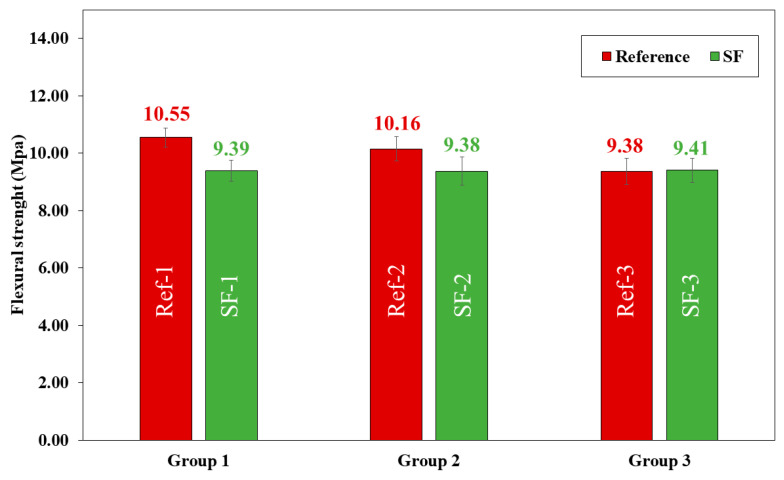
Flexural strength of all reference (Ref) and seashell filler (SF) mixtures.

The compressive and flexural strengths of mixtures are shown in Figure 10 and Figure 11, respectively, for the age of 28 days. Generically, in the reference mixtures, the compressive strength decreased as the Vp/Vs ratio increased. This confirms that the compressive strength depends on the Vp/Vs for a binary blend of powder. However, there is no general correlation between compressive strength and the Vp/Vs [28].

A decrease in compressive strength was also observed when LF was replaced by SF. These decreases were 29.43, 16.84 and 2.29% for group 1, group 2 and group 3, respectively. It can be confirmed, for this particular case, that the decrease in compressive strength due to the substitution of LF for SF is smaller as the Vp/Vs ratio increases. Considering the standard deviation found, a mixture with a Vp/Vs of 0.8 and substituting 100% LF by SF had a compressive strength similar to the reference (Ref-3 vs. SF-3).

In a generic way, these decreases could be due to several causes: (i) to particle size distribution and (ii) the SF surface texture (Figure 6 and Figure 7, respectively). SF particles have a smaller size and a less rounded shape than LF particles, which worsens the internal organization between particles, leading to a decrease in compressive strength. In addition, SF particles have a more porous surface (Figure 7) than LF particles, which worsens the interaction of cement particles with SF particles, increasing the number of pores and weakening mechanical properties. In other words, it can be said that the effective w/c ratio was lower when SF was used. Other authors have also related the shell shape, particle size distribution and higher porosity of seashells particles in hardened mortars to the decrease in compressive strength [4,19,57,62,69].

With XRD and TGA/DTA (Figure 9 and Figure 8, respectively), the results obtained indicated that a higher amount of Ca(OH)_2_ was obtained. Despite much research on the setting reactions of Portland cement, there is still no clear relationship as to whether increasing the portlandite content improves or worsens the mechanical properties [70]. Because the Ca(OH)_2_ content of the SF was very small (see TGA/DTA, Figure 5), no significant changes in phases, such as C-S-H, have been detected, or not at least for the age of 28 days with XRD and TGA/DTA.

The flexural strength showed the same trend as the compressive strength. A decrease was observed in the reference mixtures (Ref) when the Vp/Vs ratio was higher. When LF was replacing by SF, flexural strength decreased by 12.47 and 8.32% for group 1 and group 2, respectively. However, in group 3, flexural strength was the same for both Ref-3 and SF-3. 

The SF-3 mixtures are the ones that obtained similar mechanical properties to Ref-3; nevertheless, the results obtained in all mixtures are sufficiently suitable to be used in real applications. 

#### 3.4.3. Dry Bulk Density, Water Absorption Capacity and Accessible Porosity of Water

**Figure 12:**
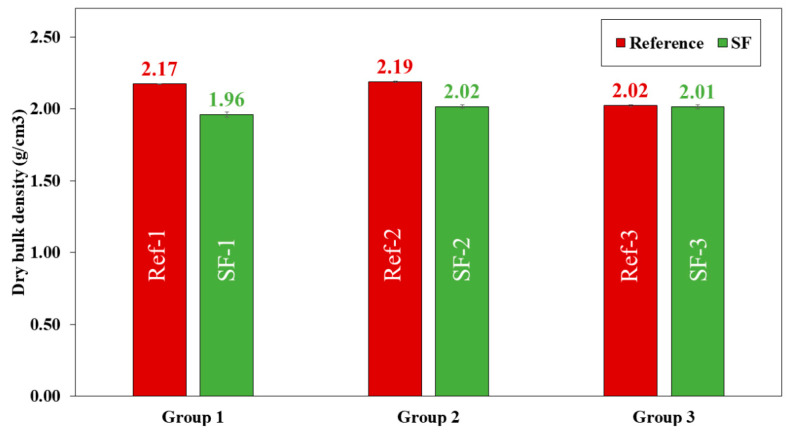
Dry bulk density (g/cm^3^) of all reference (Ref) and seashell filler (SF) mixtures.

**Figure 13:**
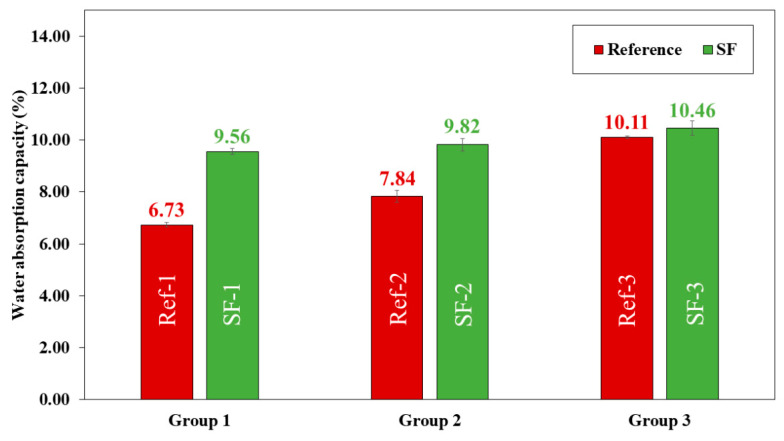
Water absorption capacity (%) of all reference (Ref) and seashell filler (SF) mixtures.

**Figure 14:**
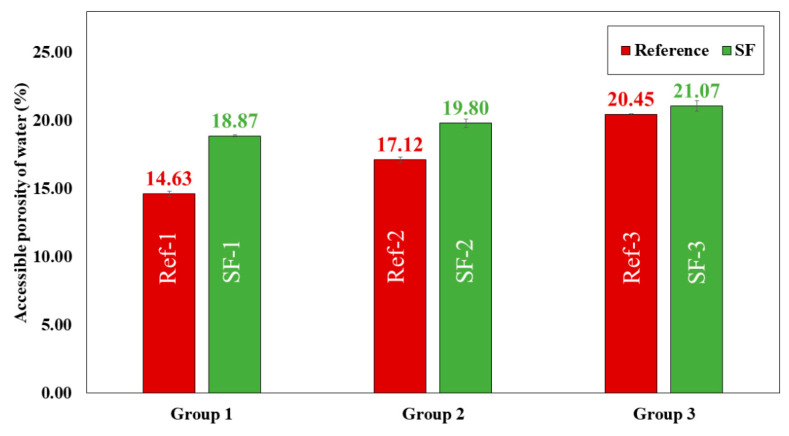
Accessible porosity of water (%) of all reference (Ref) and seashell filler (SF) mixtures.

Dry bulk density (Figure 12), water absorption capacity (Figure 13) and accessible porosity of water (Figure 14) were measurement at 28 days of curing. 

Dry bulk density (Figure 12) decreases with the substitution of LF by SF. The highest value was obtained in Ref-1 (2.17 g/cm^3^) and decreased up to SF-3 (2.01 g/cm^3^). These decreases were 10.71, 8.42 and 0.50% for group 1, group 2 and group 3, respectively. The decrease may be due to the higher number of pores formed when LF is substituted by SF, due to the irregular shape and surface texture of the shell particle, which worsens the internal organisation between particles. This is in accordance with the results obtained in compressive (Figure 10) and flexural (Figure 11) strength. Martínez-García et al. [4] also related that the shape and surface texture of mussel shell particles causes entrapped air in the concrete, hence the decrease in density. Despite the voids that are formed, the loss of density for all mixtures, especially group 3, is negligible. Due to the amount of CaO found in SF, this causes densification of the hydration products because of the increased Portlandite phase [2,12], demonstrated by TGA/DTA (Figure 8) and XRD (Figure 9) analysis.

In all mixtures, water absorption increased by replacing LF with SF. The lowest water absorption value was found in group 1 mixtures (6.73% for Ref-1 and 9.56% for SF-1). Group 2 mixtures had a value of 7.84% for Ref-2 and 9.82% for SF-2. The highest value was obtained for group 3 mixtures (10.11% for Ref-3 and 10.46% SF-3). The increase in water absorption may be related to particle size distribution (Figure 6), and irregular shape and SF surface texture (Figure 7). Higher water absorption means that more pores have been filled with water, which is related to the greater voids formed due to the worsening of the internal organisation between particles [2,10,19,62].

Accessible porosity of water (Figure 14) increases by replacing LF with SF. These increases were 22.47, 13.54 and 2.94% in group 1, group 2 and group 3, respectively. The group 3 mixtures were those with the highest accessible porosity values (20.45% for Ref-3 and 21.07% for SF-3). The increase in accessible porosity explains the decrease in compressive (Figure 10) and flexural (Figure 11) strength in all SF mixtures. This is in accordance with the loss of dry bulk density (Figure 12) and the increase in water absorption (Figure 13), also related to particle size distribution and irregular shape, and SF surface texture with the Aragonite phase present, demonstrated by XRD (Figure 9), which makes the porosity higher, as shown by G.O Bamigboye et al. [2].

In group 3 mixtures, it can be observed that for water absorption, dry bulk density and porosity there is no great difference between the reference mixtures and those containing SF. This can also be due to the packing effect that occurs when the amount of powder is very high, which causes pore filling [7].

## 4. Conclusions

The incorporation of *Acanthocardia tuberculata* shell powder as filler in self-compacting mortars production was studied. The following conclusions were reached:

- The main oxide found in *Acanthocardia tuberculata* shell powder was CaO. Aragonite and Vaterite were the main phases identified.
- An analysis by SEM was conducted in order to study the *Acanthocardia tuberculata* microstructure. A shell heated at 135 °C and an unheated shell were studied. As a result, biomineralised materials were observed in an unheated shell, which corresponded to minerals and organic macromolecules. When the shell was heated, these materials disappeared.
- The particle size distribution of *Acanthocardia tuberculata* shell powder was smaller than the limestone filler used as a reference. Both fillers were analysed by SEM, finding that *Acanthocardia tuberculata* shell powder particles were less smooth and more porous than limestone filler particles.
- Three types of mortar mixtures with 100% *Acanthocardia tuberculata* shell powder that met the parameters of self-compactability of SCM were tested. It was found that, when replacing limestone filler with *Acanthocardia tuberculata* shell powder, properties such as fluidity and consistence decreased. This is related to particle size distribution, and a more porous and less smooth surface of *Acanthocardia tuberculata* shell powder than limestone filler.
- The mineralogical phases of hardened mortars were studied using the XRD technique and TGA/DTA analysis. All mortars with *Acanthocardia tuberculata* shell powder presented the same mineralogical phases as reference mortars, except Aragonite, and increased in the Portlandite phase found in shell powder mortars. This was indicative that the presence of *Acanthocardia tuberculata* shell powder had no negative effect on cement setting, which would occur with significant changes in XRD.
- The mechanical strength was slightly reduced with the incorporation of *Acanthocardia tuberculata* shell powder. Compressive strength decreased to 29.43, 16.84 and 2.29% when replacing limestone filler by seashell filler to Vp/Vs 0.6, 0.7 and 0.8, respectively. There was a decline in flexural strength to Vp/Vs 0.6 (12.47%) and Vp/Vs 0.7 (8.42%). To Vp/Vs 0.8 was the same. Properties such a water absorption and accessible porosity of water slightly increased when *Acanthocardia tuberculata* shell powder was incorporated. These increases were 29.60, 20.16 and 3.35% to water absorption and 22.47, 13.54 and 2.94% to accessible porosity of water to Vp/Vs 0.6, 0.7 and 0.8, respectively. On the other hand, dry bulk density decreased to 10.71, 8.42 and 0.50%.
- A mixture with Vp/Vs of 0.8 and substituting 100% limestone filler with *Acanthocardia tuberculata* shell powder had similar mechanical properties to the reference mixture.

It can be concluded that the substitution of 100% limestone filler with *Acanthocardia tuberculata* shell powder as filler in a mixture with Vp/Vs of 0.8 could be effectively presented as a viable alternative to traditional limestone fillers, behaving just like a mixture made from conventional materials. In addition to decreasing the extraction of this natural material, the accumulation of seashells in landfills would be avoided.

## Figures and Tables

**Table 1:** Nomenclature, mixing proportions (kg/m^3^) and self-compactability properties of the self-compacting mortars (SCM).

		Group 1		Group 2		Group 3	
		Ref-1	SF-1	Ref-2	SF-2	Ref-3	SF-3
Powders	CEM	490.9	490.5	526.3	525.9	557.0	556.5
	Limestone filler (LF)	300.5	0	322.2	0	341.0	0
	Seashell filler (SF)	0	295.7	0	317.0	0	335.5
Fine aggregates	Fine sand (FS) (0–3)	618.1	617.6	568.1	567.6	526.0	525.6
	Coarse sand (CS) (0–6)	622.8	622.3	572.4	572.0	530.0	529.6
	Water	240.6	240.6	258.0	258.0	273.0	273.0
Self-compactability parameters	Vp/Vs	0.6	0.6	0.7	0.7	0.8	0.8
	Vw/Vp	0.85	0.85	0.85	0.85	0.85	0.85
	Sp/p %	0.6	0.7	0.6	0.7	0.5	0.6

**Table 2:** XRF chemical composition of the raw materials.

Oxides (%)	Cement (CEM)	Fine Sand (FS)	Coarse Sand (CS)	Limestone Filler (LF)	Seashell Filler (SF)
Na_2_O	0.24	−	−	0.14	0.95
MgO	1.33	0.88	37.98	0.90	0.10
Al_2_O_3_	3.73	0.20	0.06	2.50	0.15
SiO_2_	15.58	0.39	0.91	2.30	1.25
P_2_O_5_	0.09	−	−	−	−
SO_3_	4.79	0.07	0.11	0.09	0.16
Cl_2_O_3_	0.18	−	0.21	−	0.11
K_2_O	1.21	0.03	0.05	−	0.03
CaO	70.03	98.31	60.44	93.46	96.81
TiO_2_	0.23	−	−	−	−
MnO_2_	0.06	−	−	−	−
Fe_2_O_3_	2.44	0.10	0.23	0.61	0.11
ZnO	0.02	−	−	−	−
SrO	0.08	0.03	−	−	0.32

**Table 3:** Fresh properties of six SCM mixtures studied, reference (Ref) and seashell filler (SF).

	Group 1		Group 2		Group 3	
	Ref-1	SF-1	Ref-2	SF-2	Ref-3	SF-3
Gm	4.52	4.41	9.56	7.12	7.12	6.70
Rm (s^−1^)	1.25	0.91	2	1.54	1.67	1.42

## Data Availability

Not applicable.
